# Polarotactic Aggregation of the Migratory Locust *Locusta migratoria migratorioides* (Insecta: Orthoptera) Driven by Spectral-Polarization Vector Combinations

**DOI:** 10.3390/life16101610

**Published:** 2026-09-25

**Authors:** Qihang Liu, Sohaib Shahid, Linyan Zhao, Wenxi Wang, Haibo Zhong, Fen Li, Zonghan Deng

**Affiliations:** 1 School of Computer Science & Technology, Henan Institute of Science and Technology, Xinxiang 453003, China; bjliuqihang@163.com; 2 School of Breeding and Multiplication (Sanya Institute of Breeding and Multiplication), Hainan University, Sanya 572025, China; sohaibshahid90@hotmail.com (S.S.); 20243003444@hainanu.edu.cn (L.Z.); 20243009010@hainanu.edu.cn (W.W.); 20223007306@hainanu.edu.cn (H.Z.); fenli@hainanu.edu.cn (F.L.); 3 Kunming Customs Technical Center, Kunming 650228, China

**Keywords:** *Locusta migratoria migratorioides*, polarotaxis, polarotactic aggregation, linearly polarized device, circumferential vector

## Abstract

The migratory locust, *Locusta migratoria migratorioides* (Fairmaire et L.J. Reiche) (Insecta: Orthoptera) (syn. *L. m. manilensis*), exhibits polarization-sensitive (polarotactic) aggregation behavior that can be exploited for behavioral control. This study elucidates the effects of circumferential vector combination patterns generated by linearly polarized and polarization-detection violet (520 nm) + orange (610 nm) lighting systems on locust aggregation. Using standard, linearly polarized, and polarization-detection lighting devices, we quantified phototactic and polarotactic aggregation responses under controlled breeding-shed conditions to assess sensitivity differences. The results indicate that polarization characteristics are the primary determinants of differential sensitivity between polarotactic and phototactic aggregation, mediated by circumferential vector combination patterns. Light exposure duration significantly influenced aggregation responses; at 9.0 h, the polarization-detection device VI elicited the strongest polarotactic aggregation response, whereas device I induced the strongest phototactic aggregation response. Furthermore, the circumferential vector patterns produced by linearly polarized device III and polarization-detection device VI were most effective in inducing polarotaxis. These findings demonstrate that locust visual sensitivity is regulated by heterogeneous polarization properties of light and modulated by exposure duration. Leveraging the dependence of polarotactic sensitivity on specific vector combination patterns, particularly the optimal performance of device VI and the suboptimal effect of device III, at 9.0 h strategic deployment of such lighting systems can disrupt celestial polarization-based navigation. This provides a practical basis for locust behavioral control and contributes behavioral evidence on polarization-guided aggregation in locusts.

## 1. Introduction

Locust plagues are a major agricultural and ecological threat worldwide, causing severe economic losses and increasing the risk of food insecurity. Current control strategies rely predominantly on chemical insecticides, which have promoted pesticide resistance in field populations and caused damage to biodiversity [1,2]. The effectiveness of light-based physical control is limited by the phototactic and physiological response thresholds of locusts [3]. Currently, it is recognized that the high sensitivity of the dorsal rim area (DRA) of the locust compound eye to polarized light is an important mechanism for their navigation and positioning. The neural basis of polarized-light processing has been extensively studied, and the nonlinear interaction between the polarization compass and the azimuth compass in the central complex (CX) has been confirmed as an important mechanism for orientation [4]. Insect spatial orientation relies on the integration of multiple sensory modalities, including celestial polarization patterns, solar azimuth cues, visual landmarks, olfactory signals, and potentially geomagnetic fields [5]. The central complex (CX) serves as a hub for integrating these diverse inputs to generate coherent navigational outputs [6]. However, locust navigation based on celestial polarization remains vulnerable to changes in light direction, light intensity, and ecological background, which limits the practical application of polarization-based control. Therefore, improving polarized-light induction technologies and developing polarization-spectrum-based control strategies are important for advancing green pest management and supporting food security and human health.

Locust visual sensitivity arises from the integrated function of DRA and non-DRA regions, which combine spectral (wavelength), intensity, and polarization inputs. The combined effects of their color vision (light wavelength), light intensity vision, and polarization vision lead to changes in the locusts’ sensitive orientation, preference selectivity, and taxis towards polarized light. Furthermore, the influence of the polarization spectrum and its properties on the locusts’ polarization vector-sensitive response patterns is highly correlated with their visual background dependency [7,8]. Previous studies have shown that polarization-sensitive (POL) neurons encode both solar azimuth and the geometric structure of skylight polarization patterns, while also exhibiting color-opponent and spatially antagonistic responses to unpolarized light [9]. Changes in the angle of polarization (AoP) strongly modulate the physiological state of polarization-sensitive neurons, with distinct response profiles selectively tuned to specific electric field vector (E-vector) orientations. This tuning enables the locust polarization-vision system to exhibit a characteristic “weak-light enhancement and strong-light attenuation” response, thereby improving the detection of intensity, spectral composition, and contrast of polarized light signals. At the behavioral level, visual context and stimulus features dynamically reshape the internal compass representation, allowing locusts to adapt to changing environmental conditions and generate appropriate orientation responses. Consequently, structured polarization stimuli can effectively modulate locomotor orientation by influencing polarization-dependent navigation mechanisms. In addition, the spectral composition of polarized targets plays a dominant role in sensory processing and target detection, highlighting the importance of spectral–polarization interactions in shaping locust visual behavior [10].

Polarization sensitivity alone does not fully explain locust behavioral responses to light. In this study, phototactic aggregation was operationally defined as the response induced by unpolarized spectral lamps, whereas polarotactic aggregation was defined as the net response to polarized lamps above this phototactic baseline (i.e., the difference in aggregation density between polarized and unpolarized lamps). Phototactic sensitivity is largely governed by heterogeneous spectral sensitivity in non-DRA regions, with peak responsiveness typically observed in violet and orange wavelengths [11,12]. Meanwhile, the DRA’s tuning sensitivity to linearly polarized orange light and its short-range regulatory sensitivity to linearly polarized violet light demonstrate the potential applicability of the polarization opponent-sensitive output response of the locust’s polarization vision, which is induced by the coupling of long- and short-wave polarization spectrum intensities. Research on the specific response characteristics of locust polarization vision to different types of polarized light indicates that the properties of linearly polarized spectral light can modulate the locust’s vector-dependent integrated output effect [13]. The photo-induced visual sensitivity modulation effect is optimal under linearly polarized violet light, whereas the distance-related modulation effect is strongest under linearly polarized orange light. Previous studies have demonstrated that monospectral violet and orange linearly polarized light (LPL) elicits cosine-periodic tuning responses in locusts, whereas the corresponding linearly polarized detection light (LPDL) induces sinusoidal tuning responses and shifts the sensitive vector. Additionally, the polarotactic sensitivity of locusts to illumination duration exhibits a threshold effect [14]. A single linearly polarized spectrum does not alter the locust’s polarization-sensitive vector pattern, whereas a single linearly detected polarized spectrum does. Furthermore, polarization spectral properties determine the locust’s polarization-sensitive aggregation response under both linearly polarized and linearly detected polarized light. Consequently, heterogeneous polarized spectra can effectively regulate polarization-sensitive aggregation responses. In addition, sinusoidal tuning characteristics of polarization-sensitive visual acuity have been verified in both wild and laboratory locusts in response to vector patterns induced by single-spectrum polarized light (both linearly polarized and linearly detected) [15].

The current international research focus has shifted from behavioral responses to a single vector orientation toward elucidating insect navigation strategies in complex polarized environments and developing behavioral intervention technologies based on heterogeneous polarized light fields [16]. However, several aspects remain unexplored. These include leveraging identified polarization vector patterns to modulate locust visual sensitivity, determining the effects of vector combination patterns from heterogeneous polarized spectra on orientation behavior and developing practical approaches for using polarized vectorial light to disrupt locust spatial positioning within their visual background. These gaps hinder both the implementation of polarized light-based pest control strategies and a deeper understanding of polarization-dependent orientation and navigation mechanisms. Previous studies have predominantly used single-direction, single-spectrum polarized light stimulation [17], which differs considerably from the complex polarization environment in the field. Consequently, the disruptive effects of multi-directional, multi-vector combination polarized light fields on locust spatial orientation remain unexplored, and there is a lack of engineering-feasible deployment strategies for polarized light sources, specifically, whether heterogeneous spectral combinations produce synergistic or antagonistic effects, whether azimuthal multi-vector polarized light fields interfere with the polarization compass integration function of locust CX neurons, and which vector combination patterns maximize the suppression of locust spatial orientation ability-addressing; these issues are pivotal for the practical implementation of polarization-guided locust control, as well as for understanding the mechanisms underlying their polarization-dependent orientation and navigation [18].

In view of this, based on our previous studies on the polarotactic sensitivity of locusts induced by single-spectrum, single-vector linearly polarized and linearly polarized detection light, the present study further designed a violet (405 nm) + orange (610 nm) dual-spectrum azimuthally combined vector lamp. It should be clarified that the “azimuthally combined vector pattern” in this paper refers to the following: the primary structure of the lamp adopts a hexahedral structure, with each face simultaneously emitting violet or orange polarized light at specific vector angles, thereby generating a heterogeneous polarized light field with a 360° azimuthal distribution of polarization vectors within the breeding shed (Figure 1 and Figure 2, Table 1). The fundamental distinction from our earlier work on unidirectional, single-spectrum polarized light is that previously, polarized light with a single vector orientation was projected onto the locusts from one side to test their taxis toward a particular E-vectors, whereas the azimuthal lamps used in this study were placed in the center of the experimental shed as the light source center, simultaneously emitting polarized light with different vector angles in all directions to form an azimuthally heterogeneous polarized light field. Locusts in the shed were positioned around this heterogeneous field, and their overall polarotactic aggregation behavior was tested. This design more closely resembles the complex polarized light environment formed by vegetation reflection and water surface scattering in field habitats, aiming to investigate the disruptive effects of heterogeneous polarized light fields on locust spatial orientation ability rather than the inductive effects of a single polarization direction.

To date, no experimental evidence has been published regarding the use of azimuthally heterogeneous polarized light fields as a behavioral intervention strategy for locust control in either laboratory or field settings. It should be noted that the experimental design of the azimuthally heterogeneous polarized light field faced multiple constraints. Due to the different spectral properties between violet and orange light, their light inherently differs in light intensity. Similarly, linearly polarized light and linearly polarized detection light exhibit inconsistent light intensities due to different polarizer configurations. Although the illumination energy of each light source could be equalized (150 mW/cm^2^) by adjusting the LED driving current, linearly polarized detection light strictly obeys Malus’s law, meaning its intensity in each vector direction undergoes nonlinear attenuation with the change in angle, making it impossible to achieve uniform light intensity in all directions. Moreover, the behavioral responses of locusts in this environment involve the combined effects of multiple taxes, such as phototaxis and polarotaxis, thus requiring well-designed control experiments to disentangle the contribution of each factor. Finally, despite the shed environment is superior to indoor settings, it still falls short of the complex, naturally heterogeneous polarization characteristics formed by vegetation reflection and water surface scattering in the wild.

This study focuses on the specific sensitive vectors (Table 1) associated with polarization-induced aggregation in *Locusta migratoria migratorioides* (*L. migratoria migratorioides*), under heterogeneous violet and orange spectra, including both linearly polarized and linearly detected polarized light, in greenhouse environments. Utilizing custom-developed circumferential combination lamps for violet and orange light, as well as lamps with different azimuthal vector combinations of linearly and linearly detected polarized spectra, we evaluated the phototactic aggregation response of locusts to violet–orange circumferential illumination in a gauze-rearing greenhouse. In addition, we examined polarization-sensitive (polarotactic) aggregation sensitivity to different azimuthal vector combination patterns of linearly and linearly detected polarized violet + orange spectra. This study further analyzes how heterogeneous spectral properties and their associated vector combination patterns influence both phototactic and polarization-sensitive aggregation behavior, and discusses the regulatory effect of light exposure duration (4.5 h vs. 9.0 h) on locust polarotactic aggregation sensitivity. It aims to clarify the effectiveness of these azimuthal vector combination patterns in disrupting the spatial positioning of locusts in their natural habitats and to establish practical implementation methods for achieving polarization-induced aggregation using these patterns. Furthermore, this study explores the photo-induced effects of different polarization properties on locust polarotaxis and the regulatory role of azimuthal vector combination patterns. The ultimate objective is to provide a scientific basis for optimizing light-source deployment strategies for locust control using polarization spectra and to support further research on the mechanisms underlying insect polarization-sensitive visual responses.

## 2. Materials and Methods

### 2.1. Experimental Site and Insects

The experiment to measure the phototactic and polarization-sensitive aggregation effects of locusts under illumination from violet (405 nm) + orange (610 nm) spectrum combination lamps—with wavelength held constant across all treatments—as well as their various vector patterns of linearly and linearly detected polarized light was conducted from July to August 2025. Light intensity was calibrated to 150 mW/cm^2^ for all sources, though in the linear detection polarization configuration, transmitted intensity inherently varies with vector angle according to Malus’s law; this intensity–vector coupling was treated as part of the polarization stimulus rather than a confounding variable. The trial took place in a gauze-rearing greenhouse (20 × 5 × 3 m in length, width, and height) at the Fanxian Locust Rearing Base (35.80′ N, 115.49′ E) in Fan County, Puyang City, Henan Province, China. The test insects were healthy adult *L. migratoria migratorioides* reared for multiple generations in captivity and collected within one week of eclosion, with an approximately 1:1 male-to-female ratio and comparable body size (body length: 35–45 mm for males, 40–55 mm for females; visually inspected to be largely uniform across the experimental population). During the rearing period, the locusts were fed daily with fresh corn seedlings supplemented with wheat bran, under well-ventilated conditions. The natural photoperiod was approximately 14 h light/10 h dark. The temperature inside the shed was controlled at 28–32 °C, with a relative humidity of 60–70%. The rearing density was maintained at approximately 300 locusts/m^2^, consistent with that during the experimental period. Within the experimental greenhouse, the average density of the locusts was 300 locusts/m^2^, and their growth was largely uniform. Independent variables included spectral composition, polarization type, vector combination pattern, and illumination duration. The primary dependent variable was locust aggregation density within the defined sampling area.

### 2.2. Experimental Light Sources and Greenhouse Layout

Three types of violet orange lamps were constructed for the experiment: a spectral lamp used as the control, a linearly polarized spectral lamp, and a linear detection polarization spectral lamp (Figure 1). The spectra used in the experiment were violet and orange, with peak wavelengths of 405 nm and 610 nm, respectively (Figure 2). The selection of violet (405 nm) and orange (610 nm) was based on our previous studies, as reported in references [11,12], demonstrating that locust phototactic sensitivity peaks in these spectral regions, and that the two wavelengths are widely separated in the visible spectrum, allowing effective investigation of spectral–polarization coupling without confounding effects from wavelength proximity. Preliminary trials conducted in the rearing-shed environment further confirmed that other spectral bands produced considerably weaker induction effects.

Each lamp was assembled using 3W light-emitting diodes (LEDs, Hongtai Electronics Co., Ltd., Shenzhen, China). The LEDs were soldered onto 2 mm thick aluminum substrates measuring 200 × 120 mm in length and width to create 9 × 7 arrays of violet and orange spectrum modules. Three violet and three orange spectrum modules were alternately mounted on the upper and lower support structures to form a hexagonal light source body (Φ245 × 200 mm). This light source body was connected to upper and lower support plates via support columns and fixed supports, and the entire assembly was secured by support rods, creating the experimental violet and orange spectrum lamp (Φ396 × 325 mm) (Figure 1A and Figure 2A). For the linearly polarized lamp, six 2 mm thick linear polarizer sheets (210 × 130 mm, 50% transmittance, 95% polarization efficiency, PL-CIR HOYA, Tokyo, Japan) were fixed onto the upper and lower support structures around the violet and orange spectrum light source body, forming a hexagonal linearly polarized light source main body (Φ285 × 225 mm). Following the same support method as the violet and orange spectrum lamp, this assembly formed the experimental linearly polarized violet and orange lamp (Φ396 × 325 mm) (Figure 1B and Figure 2B). To create the linearly detected polarized lamp, six 0° vector linear polarizer sheets (210 × 130 mm, 50% transmittance, 95% polarization efficiency, PL-CIR HOYA, Tokyo, Japan) and six linear analyzer sheets (240 × 140 mm, 50% transmittance, 95% polarization efficiency, PL-CIR HOYA, Tokyo, Japan) were sequentially fixed from the inside out onto the support structures surrounding the violet and orange spectrum light source main body. This formed a hexagonal linear detection polarization light source main body (Φ315 × 245 mm). Following the same support method, this created the experimental linear detection polarization violet and orange lamp (Φ396 × 325 mm) (Figure 1C and Figure 2C). Based on the linear detection polarization vectors for locust polarization sensitivity in indoor and greenhouse settings, and to clarify the polarization-induced aggregation effects on locusts caused by the violet and orange vectorial light from these lamps, the specific vectors were designed. The violet–orange spectral vectors for the experimental linearly polarized lamp, and the experimental linearly polarized and linear detection polarization vectors (formed by the combination of 0° linear polarizer and linear analyzer sheets for the violet and orange spectra) are shown in Table 1. Each lamp, labeled I–VI, corresponded to a specific azimuthal vector combination pattern, with its full optical characteristics—including polarization mode (linearly polarized or linear detection polarization), spectral component (violet or orange), and the specific E-vector orientation on each of the six faces—summarized in Table 1. The non-polarized control lamp (CK) was equipped only with violet and orange spectral light sources and was not fitted with a polarizer.

The violet and orange spectrum modules were powered by a 12 V DC power supply. An illuminance meter (model: XRP-3000, resolution: 0.01 lx, Shenzhen Ouya Precision Instruments Co., Ltd., Shenzhen, China) was used to calibrate the rated illuminance of the violet and orange spectrum modules at 12 V. The measured illuminance values were 30,000 lx for violet light and 300,000 lx for orange light. Under these conditions, the irradiance of the violet and orange light sources was adjusted to the same level, 150 mW/cm^2^, using an irradiance meter (FZ-A, resolution ±5%; Beijing Instrument Co., Ltd., Beijing, China).

Based on the lamp identification numbers corresponding to different polarization vectors in the linearly polarized and linear detection polarization lamps (Table 1), the violet and orange lamps were combined and divided into two groups (7 lamps per group: Group I and Group II), which were labeled I–VI and CK (Figure 3). The experiment was conducted in seven breeding sheds (labeled i–vii), with each group of lamps being tested sequentially in each shed. For the experiment, each group of light sources was placed on a support 0.4–0.5 m above the ground and centered in the middle of its corresponding locust-rearing shed, out of seven sheds marked i–vii (Figure 3). A circular marking line with a radius of 0.8 m (approximately 2 m^2^) was set around the lamp to facilitate counting of locust aggregation.

### 2.3. Experimental Methods

For the two groups of experimental light sources (7 lamps per group, Figure 3), seven rearing sheds were used sequentially for the trials. Each day before 19:30, the seven lamps were positioned in the center of each of the seven rearing sheds (Figure 3). A circular marking line with a radius of 0.8 m was set up with the light source at its center. To avoid disturbance from the setup, the lights were turned on at 20:00 and off at 05:00 the next day. During two nighttime periods, 00:00–00:30 and 04:30–05:00, the photo-induced effects on the locusts in all seven sheds were investigated and photographed. The number of aggregated locusts within the circular marking line (an area of 2 m^2^) was recorded. Locust counts were conducted during two specific periods: 0:00–0:30 and 4:30–5:00. This selection aimed to characterize the polarotactic aggregation effects under 4.5 h and 9 h of cumulative light exposure, respectively. Preliminary experiments demonstrated that locust polarotactic aggregation density during these two intervals was significantly higher than during other periods (*p* < 0.05). Additionally, these intervals correspond to cumulative light durations of approximately 4.5 h and 9 h, respectively, and a 30 min window was required for each period to complete the photographic documentation across all seven sheds. Specifically, by the 0:00–0:30 period, the lights had been on for 4.5 h, allowing locust polarotactic behavior to stabilize; the 4:30–5:00 period, approaching light offset, reflected the peak polarotactic response following sufficient cumulative light exposure. Furthermore, these two periods excluded the initial behavioral fluctuations upon light onset and the stress-induced migration period immediately preceding and following light offset, thereby ensuring data stability and comparability. The trial for each group of lamps was completed over 7 days. After each day’s trial, and before 19:30, the seven lamps (labeled I–VI and CK) were rotated sequentially among the seven rearing sheds (labeled i–vii). This ensured that each lamp was tested once in each shed, for a total of 7 trials per lamp. Using the same method, the two groups of lamps were tested sequentially in the same seven sheds.

To distinguish polarotaxis from ordinary phototaxis, three types of lamps were compared under identical spectral conditions: the unpolarized spectral lamp (CK) served as the baseline for phototaxis, while the linearly polarized (LPL) and linear detection polarization (LDPL) lamps provided polarization information in addition to the same spectral output. Since all lamps used identical violet (405 nm) + orange (610 nm) spectral modules, any difference in aggregation between CK and polarized lamps could be attributed to the presence of polarization cues. This design allowed the net polarotactic contribution to be isolated from the phototactic baseline by subtractive comparison.

### 2.4. Data Processing and Statistics

For each experimental lamp, the mean number of locusts from the 7 trials within the circular marking line was calculated and then expressed as a ratio percentage (locust polarotactic aggregation density, locusts/m^2^) to the 2 m^2^ area (for the violet and orange lamps, this was the mean of 14 trials). To control for the effect of the initial population, the difference between this ratio percentage and the original locust distribution density in the shed (300 locusts/m^2^) was calculated for both light durations (4.5 h and 9 h), denoted as *ρ* (locusts/m^2^). Corresponding to the linearly polarized lamp, the linear detection polarization lamp, and the violet and orange lamp, their respective photo-induced aggregation effects are denoted as *ρ*_1_, *ρ*_2_, and *ρ*_3_. The differences between *ρ*_1_ and *ρ*_3_ (Δ(Delta) = *ρ*_1_ − *ρ*_3_, locusts/m^2^), and between *ρ*_2_ and *ρ*_3_ (Δ(Delta) = *ρ*_2_ − *ρ*_3_, locusts/m^2^) were calculated to compare and analyze the differences in photo-induced sensitivity between the vectorial light from the two polarized lamps and the light from the spectral lamp. The differences Δ(*ρ*_1_ − *ρ*_3_) and Δ(*ρ*_2_ − *ρ*_3_) represent the net polarotactic contribution beyond ordinary phototaxis. Positive Δ values indicate that polarization cues enhanced aggregation relative to the unpolarized spectral baseline (polarotaxis dominance), whereas negative Δ values indicate that polarization cues suppressed aggregation relative to phototaxis (phototaxis dominance). This subtractive comparison, together with the use of identical spectral modules across all lamps, effectively isolated the polarotactic effect from the phototactic baseline. For the different vectorial light conditions of the linearly polarized and linear detection polarization lamps, the locust polarization-sensitive aggregation density (*ρ*_2_ and *ρ*_3_) was used to reflect the photo-induced polarization-sensitive aggregation effect of the different vector lights under the different light durations.

Prior to all parametric statistical analyses, the normality of data distributions was assessed using the Shapiro–Wilk test (*W* > 0.90, *p* > 0.05 for all datasets), and homogeneity of variances was verified using Levene’s test (*F* < 3.50, *p* > 0.05 for all comparisons). These assumptions were satisfied for all datasets analyzed in this study. The experimental data were statistically analyzed using Excel software 2019 (Microsoft Corp., Redmond, WA, USA) and the SPSS 16.0 data processing system (SPSS Inc., Chicago, IL, USA). Further analysis of the results was conducted using custom functions in MATLAB (Version 2021a, The MathWorks, Natick, MA, USA). A one-way analysis of variance (ANOVA) was employed to analyze the degree of difference in the photo-induced locust aggregation effects under two conditions: (1) the same nighttime illumination duration but different linearly polarized and linear detection polarization vectors, and (2) different nighttime illumination durations but the same vector. This was also used to analyze the differences in the density disparity between polarization-sensitive and phototactic aggregation. Post hoc pairwise comparisons were performed using Tukey’s HSD (Honestly Significant Difference) test, with the significance level set at *p* = 0.05, to determine significant differences at the *p* = 0.05 level. Additionally, a Student’s *t*-test was used at the *p* = 0.05 significance level to analyze the significant differences between treatments with different polarization properties (linearly polarized vs. linear detection polarization) under the same nighttime illumination duration. The results are presented as mean ± standard error (SE).

To ensure that the observed aggregation responses were primarily attributable to polarization properties, several precautions were taken in the experimental design. First, all lamps (spectral control, linearly polarized, and linear detection polarization) used identical violet (405 nm) + orange (610 nm) dual-spectrum modules, so that spectral composition remained constant across comparisons of polarization modes. Second, the irradiance of all light sources was calibrated to 150 mW/cm^2^ to standardize light intensity. In linearly polarized configurations, transmitted intensity remained constant across vector directions, whereas in linear detection polarization configurations, transmitted intensity varied with vector angle according to Malus’s law—this intensity–vector coupling is inherent to the optical configuration and was treated as part of the polarization stimulus rather than a confounding artifact. Third, exposure duration was standardized at two fixed time points (4.5 h and 9.0 h) for all treatments and analyzed as an independent variable. Fourth, all lamps shared the same hexagonal structure and were placed at the same central position in each shed, ensuring consistent spatial light distribution. Fifth, net aggregation density (*ρ*) was calculated by subtracting the initial locust density from the observed induced density (Section 2.4), thereby controlling for baseline population unevenness. These design features collectively support the attribution of observed behavioral differences to polarization properties and their vector combinations, rather than to uncontrolled spectral, intensity, or spatial factors.

## 3. Results

### 3.1. Differences in Locust Polarization-Sensitive and Phototactic Aggregation Sensitivity Under Linearly Polarized and Linearly Polarized Detection Spectral Lamps Compared with Spectral Lamps

Figure 4 directly shows this net polarotactic effect by presenting the difference (Δ) between polarized lamps and the spectral control. Positive Δ values indicate polarotaxis dominance (polarization-enhanced aggregation), whereas negative Δ values indicate phototaxis dominance (polarization-suppressed aggregation). This subtractive approach effectively isolates polarotactic responses from ordinary phototaxis.

Compared with the unpolarized violet and orange spectral lamp, the linearly polarized and linearly polarized detection lamps produced treatment-specific differences between polarotactic and phototactic aggregation responses. These differences depended on polarization mode, azimuthally combined vector pattern, and exposure duration (Figure 4). Thus, Figure 4 shows whether adding polarization information enhanced or reduced aggregation relative to the spectral control.

At both 4.5 and 9.0 h, the azimuthally combined vector patterns of lamps I–VI significantly changed the difference between polarotactic and phototactic aggregation. This effect depended on polarization mode and exposure duration (linearly polarized lamps I–VI: *p* < 0.001, *F*_4.5h_ = 147.133, *F*_9.0h_ = 47.214; linearly polarized detection lamps I–VI: *p* < 0.001, *F*_4.5h_ = 86.357, *F*_9.0h_ = 12.707). Among the linearly polarized lamps, lamp III showed the strongest positive difference favoring polarization-mediated aggregation, followed by lamp VI. Among the linearly polarized detection lamps, lamp VI showed the strongest positive difference, followed by lamp V. In contrast, lamp V in the linearly polarized group and lamp I in the linearly polarized detection group produced the strongest negative differences, indicating dominance of phototactic aggregation over polarization-mediated aggregation; these were followed by linearly polarized lamp I and linearly polarized detection lamp II, respectively. For linearly polarized lamps, increasing exposure from 4.5 to 9.0 h did not significantly change the difference relative to the spectral control (*p* > 0.05). For linearly polarized detection lamps, exposure duration significantly affected this difference only for lamps I and IV (*p* < 0.05); the other lamps showed no significant duration effect (*p* > 0.05).

Overall, the direction and magnitude of the difference depended more on vector pattern than on duration alone. The positive responses observed for linearly polarized lamp III and linearly polarized detection lamp VI indicate that these vector combinations increased the contribution of polarization cues beyond the violet and orange phototactic response. Conversely, linearly polarized lamp V and linearly polarized detection lamp I appeared to suppress polarization-driven aggregation relative to phototaxis. At 9.0 h, lamp VI produced the strongest positive polarization-related difference, whereas lamp I produced the strongest negative difference. These findings suggest that some azimuthal vector combinations can strengthen aggregation through polarization cues, whereas others may interfere with the extraction of useful polarization angles.

### 3.2. Locust Polarization-Sensitive Aggregation Under Azimuthally Combined Vector Illumination from Linearly Polarized Lamps

Exposure duration significantly increased the polarotactic aggregation response to linearly polarized lamps (*p* < 0.05). Under the same exposure duration, the azimuthally combined vector pattern also had a significant effect on aggregation sensitivity (Figure 5). Figure 5 therefore shows two effects simultaneously: the ranking of vector patterns and the increase in aggregation after prolonged illumination.

At both 4.5 and 9.0 h, the vector patterns of linearly polarized lamps I–VI significantly affected locust polarization-sensitive aggregation sensitivity (Figure 5; *p* < 0.001: *F*_4.5h_ = 10.944, *F*_9.0h_ = 12.083). In both exposure durations, lamp III produced the highest aggregation response, followed by lamp VI. Increasing exposure from 4.5 to 9.0 h significantly enhanced aggregation for the same linearly polarized lamp pattern. The duration effect was strongest for lamp II (*F* = 38.211, *p* = 0.003) and weakest for lamp IV (*F* = 28.50, *p* = 0.006). Despite this increase, the rank order among vector patterns remained generally unchanged.

At 9.0 h, linearly polarized lamp III produced the highest aggregation density (1550 locusts/m^2^), followed by lamp VI (1470 locusts/m^2^). Extending exposure increased these responses by 14.81% and 14.40%, respectively. This pattern indicates that exposure duration amplifies the response, but the vector structure determines which lamp achieves the strongest aggregation. Thus, lamp III appears to provide the most effective linearly polarized vector pattern under the tested greenhouse conditions.

### 3.3. Locust Polarization-Sensitive Aggregation Under Azimuthally Combined Vector Illumination from Linearly Polarized Detection Lamps

For linearly polarized detection lamps, the effect of azimuthally combined vector illumination on polarotactic aggregation depended on exposure duration. Longer exposure significantly enhanced aggregation responses for the same lamp patterns (Figure 6). Figure 6 also indicates that the magnitude of the duration effect differed among vector combinations.

At both 4.5 and 9.0 h, the azimuthally combined vector patterns of linearly polarized detection lamps I–VI significantly affected locust polarization-sensitive aggregation sensitivity (Figure 6; *p* < 0.001: *F*_4.5h_ = 31.044, *F*_9.0h_ = 55.366). Lamp VI produced the highest aggregation response, followed by lamp V. Increasing exposure from 4.5 to 9.0 h significantly enhanced aggregation for the same linearly polarized detection lamp pattern. The duration effect was strongest for lamp III (*F* = 66.105, *p* = 0.001) and weakest for lamp V (*F* = 11.830, *p* = 0.026). However, prolonged exposure did not alter the relative ranking of the vector patterns, indicating that vector composition remained the main determinant of treatment performance.

Correlation analysis showed a significant positive relationship between the lamp vector patterns and polarotactic aggregation sensitivity under the same exposure duration. This relationship was stronger at 4.5 h, whereas the overall variation among treatments became larger at 9.0 h. ANOVA and subsequent Tukey’s HSD post hoc comparisons revealed significant differences in polarotactic aggregation sensitivity among the lamp vector patterns under the same exposure duration (*p* < 0.001). The effect was more pronounced at 4.5 h (*F* = 31.044), whereas the overall variation among treatments became larger at 9.0 h (*F* = 55.366). These results suggest that early responses are strongly associated with vector pattern, while prolonged exposure magnifies differences in aggregation intensity among the patterns.

At 9.0 h, linearly polarized detection lamp VI produced the highest aggregation density (1580 locusts/m^2^), followed by lamp V (1415 locusts/m^2^). Extending exposure increased these responses by 15.75% and 11.71%, respectively (Figure 6). Therefore, lamp VI was the most effective linearly polarized detection configuration and gave the highest aggregation density among all tested lamps.

## 4. Discussion

Spatial orientation in insects depends on the integration of spectral, intensity, and polarization cues. Previous studies have shown that locust orientation is strongly influenced by the angle of polarization (AoP) of dorsal polarized light [19,20]. The central complex (CX) integrates these inputs and contributes to compass-guided navigation. Interactions between the celestial polarization compass and the solar azimuth compass allow locusts to adjust orientation under changing environmental conditions. Therefore, a stimulus combining suitable intensity, wavelength, and E-vector information can alter locust orientation. However, responses produced by artificial polarizer stimulation should be interpreted cautiously because they may not fully reproduce the matched-filter coding of natural Rayleigh sky polarization [21].

This study demonstrates that aggregation under linearly polarized lamp III and linearly polarized detection lamp VI was significantly higher than under the spectral control, with the stronger response occurring under detection lamp VI (Figure 4). Because the receptive fields of locust CX neurons are matched to sky polarization patterns [22], the behavioral response observed here is consistent with high sensitivity to structured polarization cues. The results suggest that under specific azimuthal vector patterns, polarization information can outweigh spectral intensity information in driving aggregation. This supports the view that DRA-mediated polarization processing and non-DRA spectral processing contribute differently in complex light environments. In practical terms, polarization cues appear to act as the dominant spatial signal, whereas spectral intensity may mainly support target detection and background assessment.

The stronger response under linearly polarized detection lamp VI may be related to the coupling between light intensity and vector angle imposed by Malus’s law. Hensgen et al. reported that locust POL neurons are sensitive to changes in the degree of polarization [23]. Therefore, the nonlinear intensity attenuation produced by the detection configuration may provide additional contrast that is encoded together with the polarization angle. This could strengthen the polarotactic response relative to constant-intensity linearly polarized illumination. The matched-filter channel formed by the DRA and CX is known to support high-gain responses to polarization cues [24], which may explain the preferential aggregation around polarized light sources. For applications that require maximum attraction, the linearly polarized detection configuration therefore appears promising, although its advantage should be confirmed under field conditions.

At 9.0 h, linearly polarized lamp III and linearly polarized detection lamp VI produced statistically comparable aggregation densities (1550 vs. 1580 locusts/m^2^; *t* = 0.724, *df* = 12, *p* = 0.483) (Figure 5 and Figure 6). This suggests behavioral convergence between the two optical configurations despite their different physical inputs. Linearly polarized light mainly provides a stable E-vector cue, whereas linearly polarized detection light combines vector angle with intensity variation. Previous work has shown that locust CX neurons can integrate polarization angle and light intensity [25], and that polarization and azimuth compass signals interact nonlinearly in the CX [26]. The present behavioral convergence may therefore reflect an invariant coding process in which locusts extract polarization-angle information while partly filtering intensity fluctuations [27]. This mechanism would be advantageous in natural environments where sky polarization changes with clouds, haze, and vegetation reflections [28]. The absence of a significant difference at 9.0 h may also indicate that polarotactic aggregation approached a response plateau. Thus, when detection lamps are technically difficult to deploy, linearly polarized lamps may provide a practical alternative, especially under prolonged exposure.

Previous studies indicate that insect visual systems combine E-vector direction, wavelength, and intensity information to resolve polarization patterns [29]. In the present study, extending exposure from 4.5 to 9.0 h increased aggregation by 14.81% for linearly polarized lamp III and by 15.75% for linearly polarized detection lamp VI (Figure 5 and Figure 6). This supports a temporal accumulation effect in locust polarotactic aggregation. The result is consistent with temporal integration in CX neurons [30] and with the capacity of the locust sky compass network to integrate directional information over time [31]. Biologically, a delayed increase in response may reduce errors when polarization signals are unstable at first exposure and then become more reliable with continued stimulation. Nevertheless, this time-dependent interpretation remains a behavioral inference and should be tested with electrophysiological recordings.

The azimuthally heterogeneous vector fields, especially linearly polarized detection lamp VI, disrupted locust spatial orientation, as shown by aggregation around the light source rather than a uniform distribution. A plausible explanation is cue conflict within the CX. Under natural conditions, sky polarization and solar azimuth cues usually combine to produce a coherent directional output. In the present lamp field, different regions of the compound eyes likely received polarization cues from different directions, which could activate POL neurons with competing preferred directions and reduce the dominance of any single compass signal [32]. The light source may then become the most stable spatial reference, leading to local aggregation. This interpretation is consistent with reports that the insect central complex integrates multiple navigation cues [33] and that heterogeneous polarized input can influence modality-specific visual circuits [34]. However, because neural activity was not recorded here, the proposed CX conflict mechanism should be considered a mechanistic hypothesis rather than direct evidence.

Not all vector combinations produced the same behavioral outcome. Some patterns enhanced polarotactic aggregation, whereas others reduced it relative to phototaxis. This indicates that the effect of an azimuthal vector field depends on both polarization mode and vector arrangement, and it supports the idea that locusts process linearly polarized and linearly polarized detection light through partially distinct sensitivity mechanisms [35]. These results also highlight the importance of integrating heterogeneous polarization spectra, vector patterns, and polarization properties [36]. The strong response to linearly polarized detection lamp VI (violet spectrum: 240°, 270°, 180°, 0°; orange spectrum: 30°, 240°, 90°) may arise because this pattern interferes with matched-filter coding of natural sky polarization in CX neurons [37,38]. From an applied perspective, the aim should not only be to guide locusts with a single polarization direction but also to create a polarization trap that disrupts autonomous orientation and concentrates insects near the light source. Such a strategy could reduce escape behavior, although its persistence and possible habituation require field validation.

The above discussion on behavioral disruption naturally raises a practical question: can these greenhouse effects be translated into economically viable field applications? When considering the application potential of polarized light technology, it should be acknowledged that locusts exhibit strong phototaxis to conventional visible light sources—a well-established fact widely utilized in current trapping practices. Therefore, this study does not propose polarized light as a replacement for conventional sources, which would be neither realistic nor necessary, but rather as an enhancement strategy—adding a polarization vector dimension to existing light-trapping systems to achieve greater behavioral regulatory efficiency under comparable energy input.

Our data demonstrate that under the optimal vector combination (lamp VI), polarized spectral lamps induced aggregation densities approximately 66–86% higher than non-polarized spectral controls, representing a substantial efficiency gain for the addition of a polarized optical system. Moreover, the unique “orientation disruption” effect—interfering with locust polarization compass integration through heterogeneous vector fields—provides a behavioral regulatory function that conventional light sources cannot offer, and this may translate into higher capture rates and lower escape rates under field conditions.

Nevertheless, these conclusions are based on greenhouse data and have not yet been validated in open-field environments. In natural settings, factors such as background polarized light, vegetation reflection, meteorological conditions (wind speed, cloud cover, etc.), and spatial heterogeneity of locust distribution may all influence device performance. Thus, the ultimate assessment of economic feasibility must rely on field-comparative trials, including long-term monitoring of equipment costs, energy consumption, capture efficiency, maintenance requirements, and bycatch rates of non-target insects.

Based on the current evidence, we cautiously consider that polarized light components, deployed as enhancement modules for conventional trapping systems, hold clear application potential—particularly in locust management scenarios that demand high trapping efficiency and behavioral disruption. Whether this potential translates into economically “worth it” outcomes in field practice, however, awaits further investigation.

While our results demonstrate that azimuthally heterogeneous polarized light fields significantly affect locust aggregation, we acknowledge that spatial orientation in natural environments involves multiple interacting cues. Magnetic fields, spatial geometry, and other environmental parameters may also influence locust orientation behavior [39,40]. The present study was deliberately designed to isolate the effect of polarized light cues under controlled conditions, and therefore does not account for these additional factors. Future studies should investigate how polarized light interacts with other orientation cues in more complex, ecologically realistic settings.

Although this study clarifies how azimuthal vector combinations of linearly polarized and linearly polarized detection light regulate locust polarotactic aggregation, several limitations remain. First, only healthy adult locusts within one week of emergence were tested, so age, sex, and physiological condition were not fully assessed. Second, the experiments were conducted under shed conditions; background polarization, wind, vegetation reflection, and other field factors may alter the response. Third, light intensity varied among vector directions in the detection lamps according to Malus’s law, and the violet and orange components also differed in intensity. Although the irradiance of the light sources was adjusted to 150 mW/cm^2^, polarization-vector effects cannot be completely separated from intensity effects. Finally, aggregation density was the only behavioral endpoint. Individual movement trajectories, residence time, and turning behavior were not recorded, so active polarotaxis cannot be fully distinguished from passive retention or random walking. Further electrophysiological recording, trajectory tracking, and field trials are needed to confirm the proposed mechanism.

Despite these limitations, this study provides a clearer behavioral basis for using azimuthally heterogeneous polarized light fields in locust management. It shows that the response is jointly regulated by polarization mode, vector combination, and exposure duration, with linearly polarized lamp III and linearly polarized detection lamp VI producing the strongest aggregation under the tested conditions. The findings support, but do not by themselves prove, the involvement of DRA/non-DRA division of labor and CX-based nonlinear integration. The identified vector combinations and exposure thresholds can guide engineering design of intelligent polarized-light traps, while future work should test their stability and selectivity under open-field conditions.

## 5. Conclusions

This study used violet (405 nm) and orange (610 nm) dual-spectrum lamps with unpolarized, linearly polarized, and linearly polarized detection configurations to examine how azimuthally combined vector patterns regulate polarotactic aggregation in *L. migratoria migratorioides* under greenhouse conditions. The results show that polarization mode and vector arrangement strongly determine aggregation performance. Linearly polarized lamp III and linearly polarized detection lamp VI induced higher aggregation than the unpolarized spectral control, whereas linearly polarized lamp V and linearly polarized detection lamp I produced weaker polarization-related responses. This indicates that behavioral induction depends on the selected E-vector combination rather than on the presence of polarization alone. Prolonged illumination from 4.5 to 9.0 h increased aggregation intensity, but it did not change the relative ranking of the most effective vector patterns. At 9.0 h, linearly polarized lamp III (1550 locusts/m^2^) and linearly polarized detection lamp VI (1580 locusts/m^2^) showed comparable induction efficacy, suggesting that locusts can extract polarization-angle information despite differences in intensity and vector coupling. Because this conclusion is based on behavioral aggregation data, the involvement of DRA/non-DRA processing and CX-based integration should be interpreted as a plausible mechanism rather than direct neural evidence. Overall, the findings support the use of azimuthally heterogeneous polarized light fields, especially linearly polarized detection lamp VI, as a potential strategy for disrupting locust spatial orientation and concentrating individuals near light sources. Future work should combine field validation, trajectory tracking, and neurophysiological recordings to confirm the mechanism and assess operational stability, selectivity, and long-term performance.

## Figures and Tables

**Figure 1:**
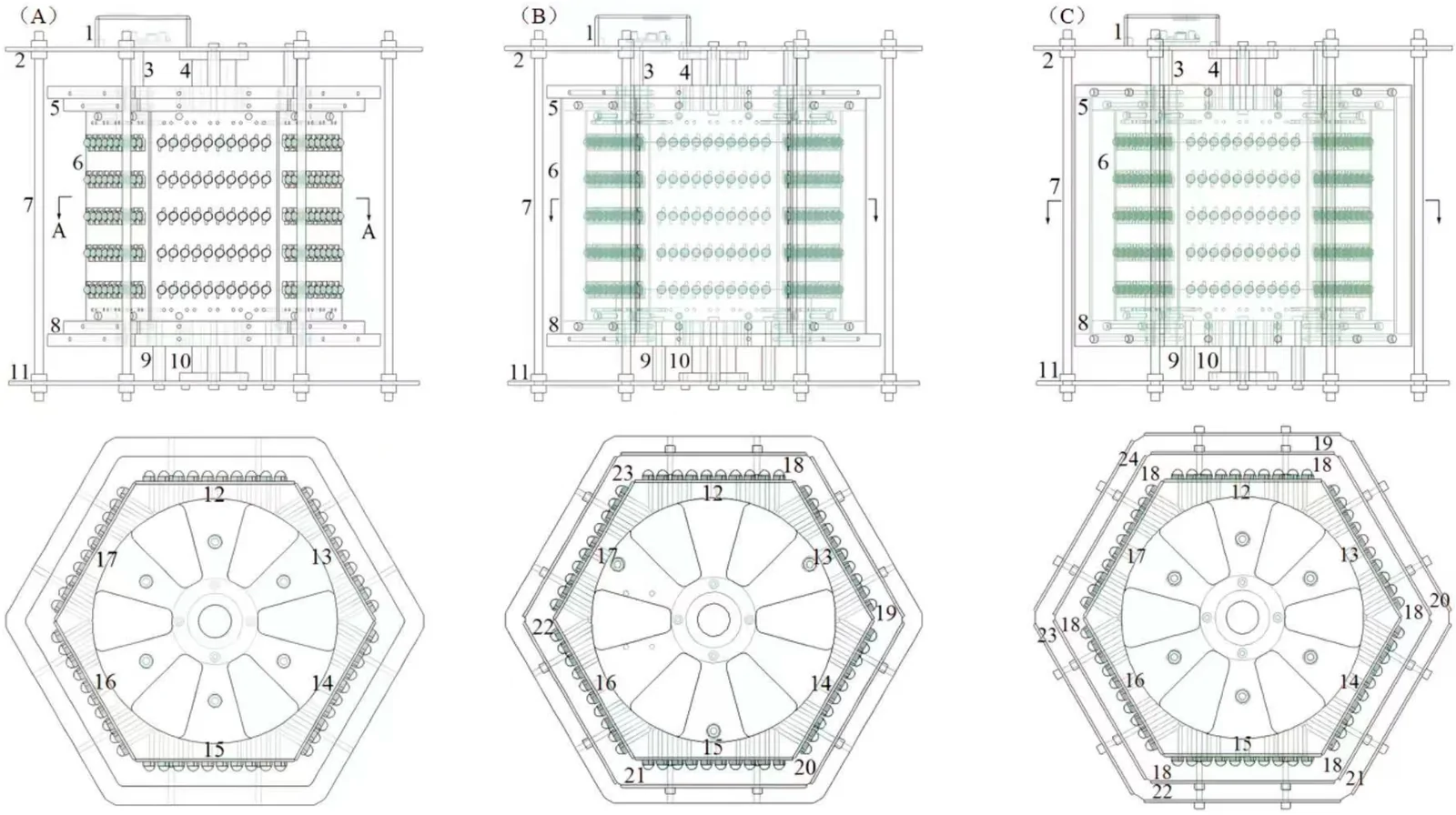
Design model of the experimental lamp. (**A**) Model of the spectral (violet (405 nm) and orange (610 nm)) lamp. (**B**) Model of the linearly polarized spectral (violet and orange) lamp. (**C**) Model of the linearly detected polarized spectral (violet and orange) lamp. 1. DC power supply (12 V); 2. upper support plate; 3. upper support column; 4. upper fixed support; 5. upper support frame; 6. light source body; 7. support rod; 8. lower support frame; 9. lower support column; 10. lower fixed support; 11. lower support plate; 12, 14, 16. violet spectrum LEDs; 13, 15, 17. orange spectrum LEDs. Linearly polarized violet and orange lamp: 18–23. Linear polarizers. Linearly detected polarized violet and orange lamp: 18.0° vector linear polarizer, 19–24. Linearly detected polarizer. A–A, cross-sectional view.

**Figure 2:**
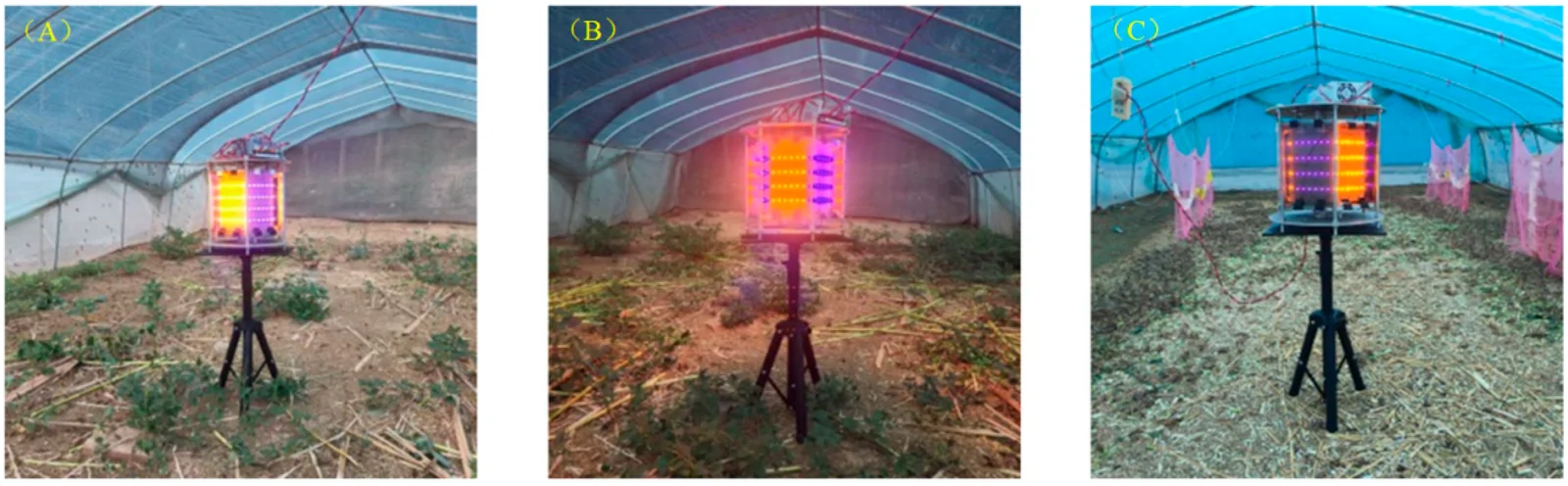
Spectra and arrangement of the experimental lamps. (**A**) Spectral (violet 405 nm) and orange 610 nm) lamp (CK). (**B**) Linearly polarized spectral (violet and orange) lamp. (**C**) Linear detection polarization spectral (violet and orange) lamp.

**Figure 3:**
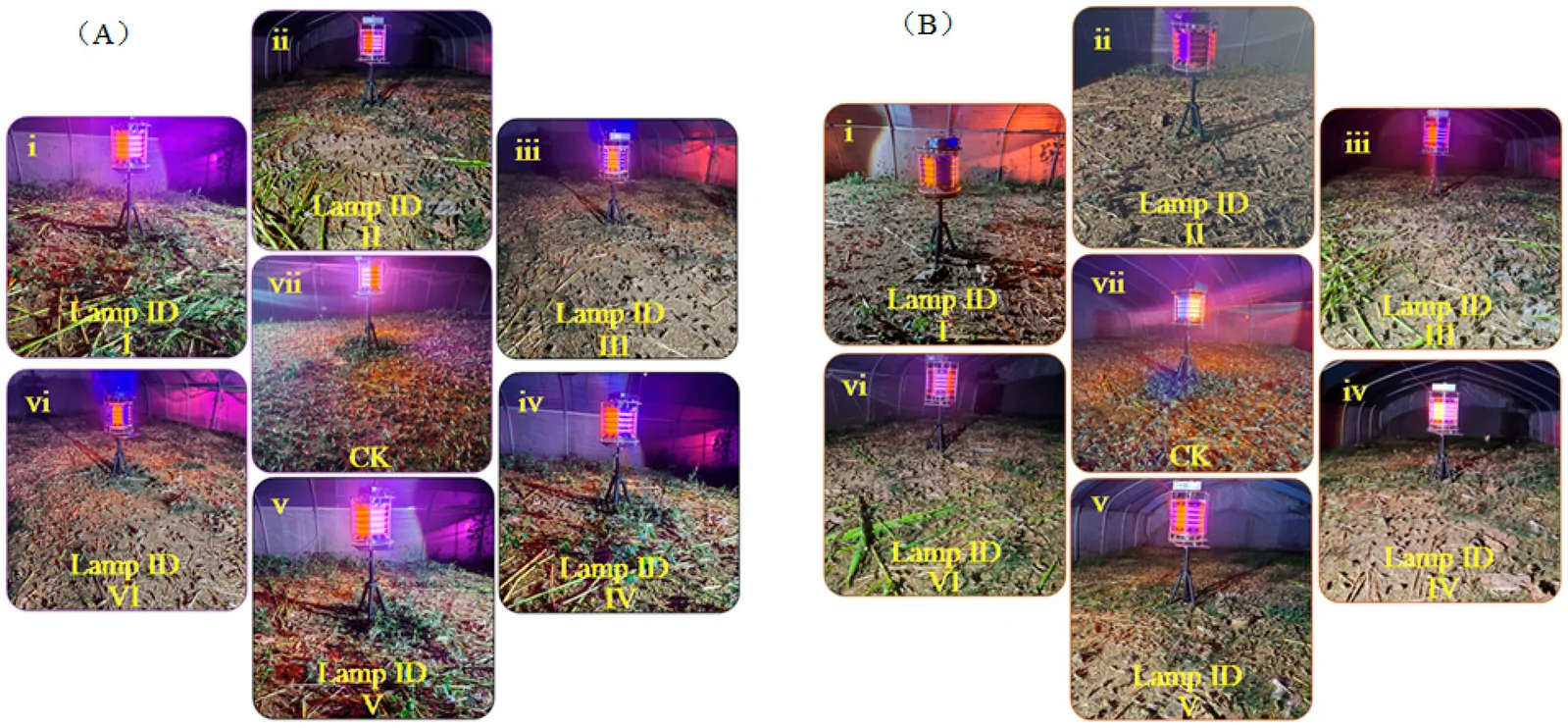
Arrangement of the experimental lamps in a greenhouse and their effects. (**A**) Group I: linearly polarized and spectral lamps. (**B**) Group II: linear detection polarization and spectral lamps.

**Figure 4:**
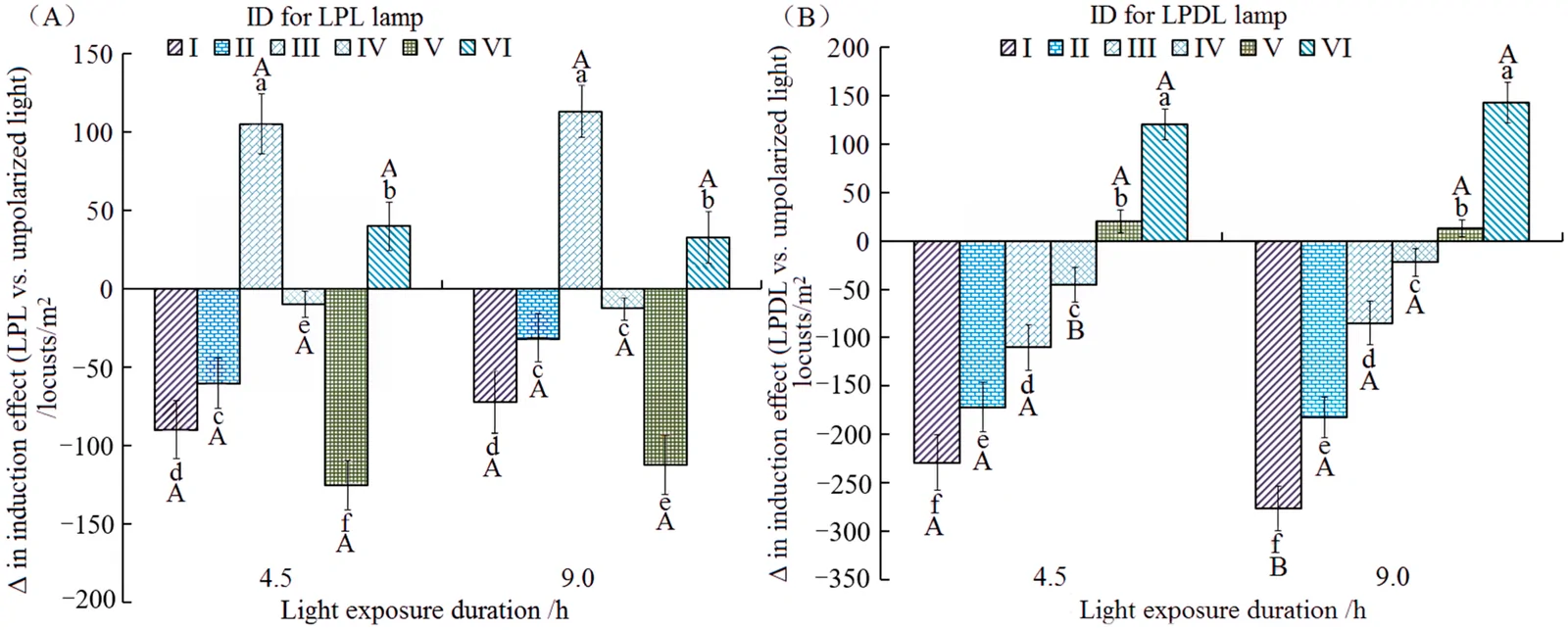
Differences in the gap between polarization-sensitive and phototactic aggregation densities of locusts under various lamp types. (**A**) Linearly polarized spectral lamp and spectral lamp. (**B**) Linear detection polarization spectral lamp and spectral lamp. Note: Within the same illumination duration, different lowercase letters indicate significant differences (*p* < 0.05, Tukey’s HSD) in the locust polarization-sensitive and phototactic aggregation densities among the different circumferential combined vectors of linearly polarized and linear detection polarization lamps (indicated by labels I–VI); the same lowercase letter indicates no significant difference (*p* > 0.05, Tukey’s HSD). For the same circumferential combined vector (i.e., the same lamp label), different uppercase letters indicate significant differences (*p* < 0.05, Tukey’s HSD) in the locust polarization-sensitive and phototactic aggregation densities between different illumination durations; the same uppercase letter indicates no significant difference (*p* > 0.05, Tukey’s HSD).

**Figure 5:**
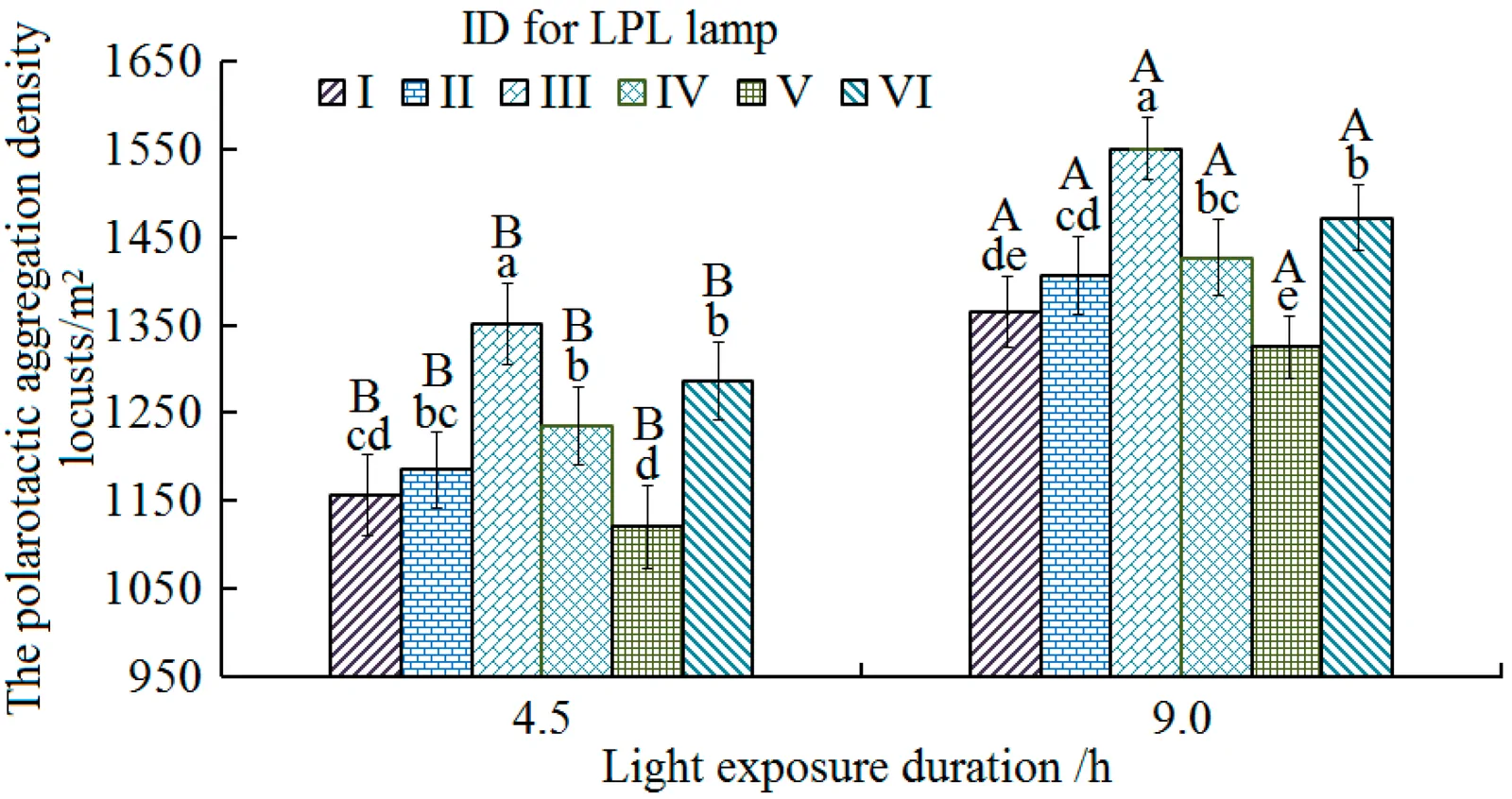
Polarization-sensitive aggregation sensitivity of locusts to the vector-combined circumferential illumination from different linearly polarized lamps. Note: Within the same illumination duration, different lowercase letters indicate significant differences (*p* < 0.05, Tukey’s HSD) in locust polarization-sensitive aggregation density among the different circumferential combined vectors of the linearly polarized lamps (indicated by labels I–VI); the same lowercase letter indicates no significant difference (*p* > 0.05, Tukey’s HSD). For the same circumferential combined vector (i.e., the same lamp label), different uppercase letters indicate significant differences (*p* < 0.05, Student’s *t*) in locust polarization-sensitive aggregation density between different illumination durations; the same uppercase letter indicates no significant difference (*p* > 0.05, Student’s *t*).

**Figure 6:**
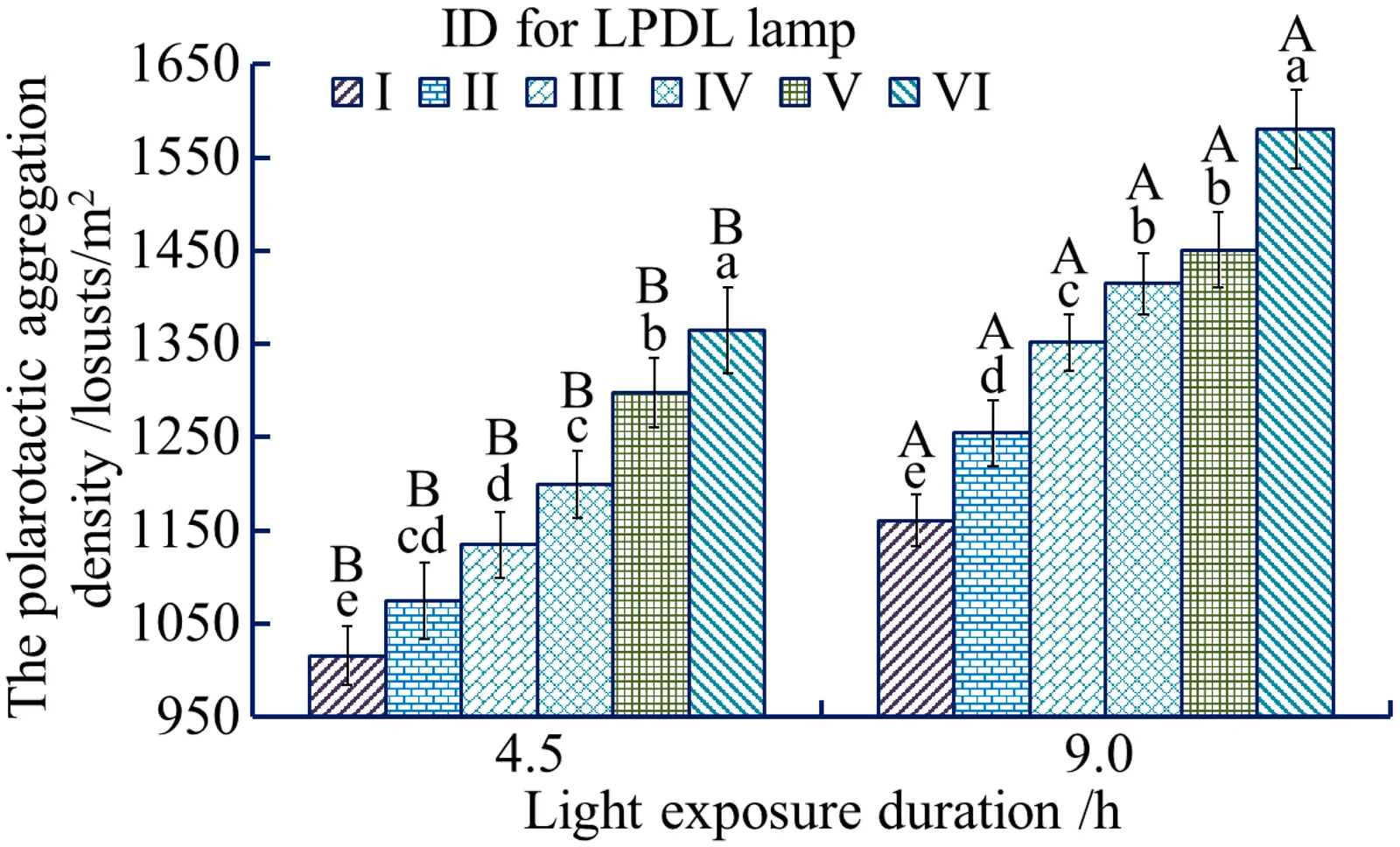
Sensitivity of locusts’ polarization-sensitive aggregation in response to vector-combined circumferential illumination from various linear detection polarization lamps. Note: Within the same illumination duration, different lowercase letters indicate significant differences (*p* < 0.05, Tukey’s HSD) in locust polarization-sensitive aggregation density among the different circumferential combined vectors of the linear detection polarization lamps (indicated by labels I–VI); the same lowercase letter indicates no significant difference (*p* > 0.05, Tukey’s HSD). For the same circumferential combined vector (i.e., the same lamp label), different uppercase letters indicate significant differences (*p* < 0.05, Student’s *t*) in locust polarization-sensitive aggregation density between different illumination durations; the same uppercase letter indicates no significant difference (*p* > 0.05, Student’s *t*).

**Table 1:** Experimental vector angles (°) for the violet and orange spectra of linearly polarized and linear detection polarization lamps.

Lamp Type		Linearly Polarized						Linear Detection Polarization					
Lamp ID		I	II	III	IV	V	VI	I	II	III	IV	V	VI
Spectrum	Violet	0	0	0	30	270	270	0	30	240	240	240	240
	Orange	180	270	90	180	0	0	30	180	0	30	30	180
	Violet	120	180	30	120	180	180	90	120	180	270	270	270
	Orange	270	0	180	270	270	270	240	270	90	240	240	0
	Violet	180	90	120	180	0	90	180	180	0	0	180	180
	Orange	90	180	270	90	180	180	90	90	30	180	90	270

## Data Availability

The original contributions presented in this study are included in the article. Further inquiries can be directed to the corresponding author.
